# Exploring antecedents and consequences of value co-creation of women entrepreneurs: a conceptual framework and research agenda

**DOI:** 10.3389/fpsyg.2026.1569735

**Published:** 2026-10-05

**Authors:** Bello Hassan, Bahtiar Mohamad

**Affiliations:** Othman Yeop Abdullah Graduate School of Business (OYAGSB), Universiti Utara Malaysia, Kuala Lumpur, Malaysia

**Keywords:** individual dynamic capabilities, opportunity recognition, entrepreneurial orientation, entrepreneurial networks, value co-creation, women entrepreneurs

## Abstract

This study examines the antecedents and outcomes of value co-creation among women entrepreneurs, emphasizing the importance of addressing the unique challenges they encounter in entrepreneurial activities. While prior studies have investigated women’s entrepreneurial motivations and barriers, the concept of value co-creation in this context remains insufficiently explored, thereby limiting theoretical development in the field. This conceptual paper develops a model derived from an extensive, critical, and multidisciplinary literature review. The review synthesizes insights from entrepreneurship, management, and network theory to explain how individual dynamic capabilities, opportunity recognition, entrepreneurial orientation, and entrepreneurial networks collectively foster value co-creation among women entrepreneurs. The study identifies four key antecedents of value co-creation: individual dynamic capabilities, opportunity recognition, entrepreneurial orientation, and entrepreneurial networks. It also proposes that value co-creation positively influences business performance. The study introduces an integrative conceptual framework and formulates five testable hypotheses to guide future empirical research. The paper provides theoretical and practical insights for policymakers and entrepreneurship support organizations to enhance women’s entrepreneurial success through value co-creation strategies.

## 1 Introduction

The concept of value co-creation has become a popular business strategy in both business-to-business (B2B) and business-to-consumer (B2C) contexts for understanding how companies work with stakeholders to facilitate the flow of information, knowledge, and resources to develop personalized products and services that meet customers’ needs (Zhang and Chen, 2008; Park and Lee, 2015; Buhalis and Sinarta, 2019). Value co-creation is a collaborative process in which stakeholders jointly create a unique experience of value (Prahalad and Ramaswamy, 2004). As researchers continue to explore the field, the concept expands beyond the service-dominant logic into areas like marketing, open innovation, and entrepreneurship (Chesbrough, 2003; Howe, 2006; Vargo et al., 2008; Leclercq et al., 2016; Shams and Kaufmann, 2016; Misiak-Kwit et al., 2021).

However, there is a critical need to adopt a gender-specific lens on value co-creation to better understand the distinct relational, empathetic, and community-oriented approaches that women bring to entrepreneurial collaboration. Women entrepreneurs often operate within distinct social, cultural, and institutional constraints that shape their entrepreneurial behavior and co-creation practices (Ahl, 2006; Marlow and McAdam, 2013). For women entrepreneurs, value co-creation is vital for their business growth. It emphasizes a collaborative approach where stakeholders collaborate to co-create value (Vershinina et al., 2020). Unlike their male counterparts, women entrepreneurs frequently employ relational, empathetic, and community-oriented approaches that align with feminist theories of entrepreneurship, emphasizing cooperation over competition and shared success over individual gain (Calás et al., 2009; Eddleston and Powell, 2008). Women entrepreneurs, often leveraging their unique experiences and leadership styles, play a significant role in fostering inclusive and diverse ecosystems that promote innovation, adaptability, and sustainability (Vershinina et al., 2020; Audretsch et al., 2020).

This feminist-informed perspective strengthens the theoretical rationale for examining value co-creation through a gender lens, as it highlights how women’s relational strategies and communal orientations shape the co-creation process. Importantly, value co-creation functions as a growth mechanism that enables women entrepreneurs—particularly those managing early-stage ventures or small and medium enterprises (SMEs)—to overcome resource constraints by mobilizing external knowledge, partnerships, and customer input. Through co-creation, they can access shared resources and expertise, thereby enabling business growth by improving product–market fit, strengthening customer loyalty, and accelerating innovation cycles (Payne et al., 2008). This strategic engagement transforms limited internal capacity into collective capability, allowing women entrepreneurs to operate more competitively within dynamic market environments.

Empirical studies have consistently shown that women entrepreneurs actively customize products and services using customer feedback and collaborative design, especially during the early stages of their businesses. This strategy often responds to resource constraints and market uncertainty, allowing women entrepreneurs in small and medium enterprises (SMEs) to co-create value with their customers. Studies indicate that women entrepreneurs depend on informal market testing, customer conversations, and social networks to refine their offerings and ensure a good market fit. Through these interactive processes, entrepreneurs gather real-time insights that influence product features, packaging, and service delivery, embodying a bottom-up innovation approach.

Global Entrepreneurship Monitor reports that the global average TEA rate for women entrepreneurs was 10.4 percent, compared to 13.6 percent for men, indicating a gender parity gap. Latin America and the Caribbean had the highest women’s TEA rates, at 30.4 and 24.1 percent, compared to Europe, at 7.8 and 6.1 percent, respectively (Hill et al., 2022). This performance attracted the attention of researchers and policymakers in recent years (Banu et al., 2021; Ali et al., 2021). In both developed and developing economies, the number of women entrepreneurs increased yearly (MIWE, 2020; Hill et al., 2022). However, despite this growth, women entrepreneurs face disproportionately high failure rates, with nearly 50% of women-led enterprises in developing economies failing within the first 3 years due to structural, financial, and network-related barriers (World Bank, 2019; IFC, 2020; UN Women, 2021). This alarming trend underscores the importance of understanding how value co-creation can serve as a strategic mechanism to enhance resilience, sustainability, and competitiveness among women entrepreneurs.

Many women entrepreneurs struggle to grow their businesses beyond subsistence levels, which serves as a key driver of their innovation and growth (Hashimu, 2018; World Bank, 2019; Nair, 2020; Audretsch et al., 2022). Previous research has identified several factors that promote value co-creation among women entrepreneurs’ businesses. Among the most common are individual dynamic capabilities (Eisenhardt and Martin, 2000; Zahra et al., 2006; Katkalo et al., 2010), opportunity recognition (Shamsudeen et al., 2017; Khin and Lim, 2018; Shepherd and Patzelt, 2018), entrepreneurial orientation (Miller, 2011; Ribeiro et al., 2021), and entrepreneurial networks (Udimal et al., 2021; Welsh et al., 2018). These factors offer valuable insights into studying value co-creation at the individual level. While research on factors that drive women to become entrepreneurs has been one of the most studied areas by researchers (Cabrera and Mauricio, 2017; Anderson and Ojediran, 2022), the literature remains limited and insufficient regarding value co-creation among women entrepreneurs.

Additionally, there is a need to empirically test the relationships between the constructs in most existing studies (Gbadamosi, 2019; Nair, 2020; Welsh et al., 2018; Misiak-Kwit et al., 2021; Audretsch et al., 2022). Therefore, this paper provides insight into the literature on value co-creation, with a specific focus on the context of women entrepreneurs, by developing research based on individual dynamic capabilities, opportunity recognition, entrepreneurial orientation, and entrepreneurial networks in relation to the value co-creation of women entrepreneurs.

This study aims to:

1. Identify and synthesize the key antecedents and consequences of value co-creation for women entrepreneurs.
2. Propose an integrative conceptual framework linking these constructs
3. Develop a set of testable hypotheses and a future research agenda to guide empirical inquiry.

The paper proceeds with a review of the literature on value co-creation and women entrepreneurship, followed by the development of our hypotheses and conceptual framework. We then present a research agenda and discuss the theoretical and practical implications of our study.

## 2 Review methodology

This study employs a narrative-conceptual review methodology to synthesize existing literature and develop a theoretical framework. Unlike a systematic review, which aims for an exhaustive and replicable search to aggregate findings, a narrative-conceptual review is ideal for providing a comprehensive, critical, and interdisciplinary perspective on a developing field, thereby constructing a theoretical foundation for future research (Baumeister and Leary, 1997; Ferrari, 2015). This approach is particularly suited to our objective of integrating insights from entrepreneurship, management, and marketing to explore the antecedents and consequences of value co-creation for women entrepreneurs, a domain where established models are still emerging.

The literature search was conducted using major academic databases, including Scopus, Web of Science, and Google Scholar, to ensure a robust coverage of high-impact and niche publications. The search strategy utilized a combination of keywords and Boolean operators centered on the core constructs of the study. Primary search terms included: “value co-creation” OR “co-creation” AND “women entrepreneur” OR “female entrepreneur.” These were subsequently combined with terms related to the identified antecedents: “individual dynamic capabilities,” “opportunity recognition,” “entrepreneurial orientation,” and “entrepreneurial networks.” The search was initially focused on literature published between 2000 and 2024 to capture the modern evolution of these concepts, but seminal works predating this period (e.g., Prahalad and Ramaswamy, 2004; Teece, 2007) were also included for foundational context.

The selection of literature was guided by pre-determined inclusion and exclusion criteria to maintain relevance and scholarly rigor. Included were: (1) peer-reviewed journal articles and seminal books; (2) publications in the fields of business, management, entrepreneurship, and marketing; and (3) both empirical and conceptual papers that provided insights into the defined constructs, with a specific emphasis on studies involving or applicable to women entrepreneurs. Excluded were: (1) non-peer-reviewed articles, editorials, and conference abstracts (unless highly influential); (2) studies not published in English; and (3) literature that did not explicitly connect to the framework’s core variables. The snowballing technique, reviewing the reference lists of key articles, was also employed to identify additional relevant sources (Greenhalgh and Peacock, 2005).

The analytical approach was thematic and synthesis-oriented. The identified literature was analyzed to distill central themes, definitions, and relational dynamics around each construct (i.e., value co-creation, individual dynamic capabilities, etc.). This involved a process of iterative reading and categorization, where findings from disparate studies were compared and integrated to identify consensus, contradictions, and critical gaps (Braun and Clarke, 2006). The synthesis was structured around explaining the nature of each construct, its specific relevance to women entrepreneurs, and the theoretical logic linking it to value co-creation and business performance. This analytical process directly informed the development of the summary tables (Tables 1–4) and the formulation of the five testable hypotheses, culminating in the proposed conceptual framework presented in Figure 1.

**Table 1:** Summary of definitions of value co-creation and their relevance to women entrepreneurs.

Author(s) and year	Definition of value co-creation	Key focus/perspective	Relevance to women entrepreneurs
Prahalad and Ramaswamy (2004)	Value co-creation is the process by which consumers and firms collaboratively create value through dialogue, interaction, and shared experiences.	Interactional and experiential; emphasizes customer engagement.	Women entrepreneurs often rely on close, trust-based customer interactions to co-develop products and services, particularly in sectors that are relationship-driven.
Vargo and Lusch (2004)	Value is co-created jointly by firms and customers through resource integration and service exchange rather than being produced solely by firms.	Service-Dominant Logic (S-D Logic) emphasizes the integration of resources.	Women entrepreneurs typically leverage social and relational resources such as empathy and community ties to co-create value through networks and partnerships.
Payne et al. (2008)	Co-creation involves linked processes between the customer, the provider, and encounters that together shape the co-creation experience.	Process-based; emphasizes interactions and mutual learning.	Women entrepreneurs frequently engage customers in iterative feedback and adaptive design processes, refining offerings through experiential learning.
Grönroos (2011)	Co-creation occurs when firms and customers jointly influence the formation of value, extending beyond traditional transactions.	Relational and interactional; emphasizes value-in-use.	Women entrepreneurs often focus on long-term relationships and value-in-use through personalized offerings that reflect customer lifestyles and preferences.
Ranjan and Read (2016)	Co-creation involves both co-production (joint activities) and value-in-use (customer experience of value).	Dual-component model integrating process and outcome.	Women entrepreneurs engage customers not just in production but also in experiential value creation, such as co-developing meaning and brand identity.
Chathoth et al. (2013)	Co-creation is the active participation of customers in designing, delivering, and improving service experiences.	Customer participation and experiential innovation.	Women entrepreneurs often adopt participatory design approaches, utilizing customer input to refine their services, particularly in the hospitality, fashion, and craft industries.

**Table 2:** Summary of the relationship between opportunity recognition and value co-creation of women entrepreneurs.

Author(s) and year	Dimension	Opportunity recognition	Link to value co-creation
Hills et al. (1997)	Customer Insight and Needs Identification	Women entrepreneurs often use close relationships and empathy to identify unmet customer needs and social problems as new opportunities.	Co-creation begins when entrepreneurs engage customers to refine identified needs into viable offerings.
DeTienne and Chandler (2007); Al-Dajani and Marlow (2013)	Networking and Social Interaction	Women entrepreneurs rely heavily on social and relational networks to sense and evaluate opportunities.	These networks offer co-creation platforms where partners collaborate to develop solutions and share resources.
Shane and Venkataraman (2000); Sarasvathy (2008)	Learning and Experimentation	Opportunity recognition emerges through experiential learning, feedback, and iterative experimentation.	Continuous interaction with customers fosters co-learning, shared design, and innovation.
Kirkwood (2009); Carter et al. (2015)	Innovation Orientation	Women entrepreneurs often transform identified gaps into innovative products or services.	Collaborative innovation enhances value propositions through mutual input and shared creativity, fostering a more effective approach to value creation.
Henry et al. (2016); Panda (2018)	Social and Community Value Creation	Opportunities are frequently identified in community-oriented or socially impactful contexts.	Co-creation activities align with social goals, enhancing both business and community value.

**Table 3:** Summary of the relationships between entrepreneurial orientation and value co-creation of women entrepreneurs.

Author(s) and year	Key EO dimensions examined	Value co-creation aspect	Findings/relationship	Implications for
Lumpkin and Dess (1996)	Innovativeness, Proactiveness, Risk-taking	Customer engagement and innovation	EO fosters experimentation and customer collaboration that leads to co-created innovative offerings.	Women entrepreneurs can leverage innovative thinking and proactive engagement to strengthen customer-based co-creation.
Teece (2007)	Strategic adaptability and opportunity recognition	Co-creation through dynamic capabilities	EO enhances sensing and seizing opportunities for joint value creation.	Encourages women to align EO with adaptive strategies that facilitate mutual learning and innovation with customers.
Kraus et al. (2019)	Autonomy, Competitive aggressiveness	Collaborative partnerships and customer trust	High EO promotes partnership building and long-term customer collaboration.	Women Entrepreneurs with strong EO can build relational trust and collective innovation with stakeholders.
Welsh et al. (2018)	Innovativeness and Proactiveness	Digital co-creation and social value	EO enhances women’s ability to utilize digital tools for participatory innovation.	Women entrepreneurs use EO to create inclusive, community-oriented value through digital co-creation.
Ndubisi and Nair (2009)	Risk-taking and Proactiveness	Customer relationship co-development	EO dimensions predict commitment to customer-centric innovation.	Women entrepreneurs co-develop solutions with customers by combining EO traits with empathy and responsiveness.
Satar and John (2019)	Innovativeness and Proactiveness	Market-driven value creation	EO leads to the identification of customer needs and joint problem-solving.	Women entrepreneurs apply EO to align co-created solutions with evolving customer expectations.

**Table 4:** Summary relationship between entrepreneurial networks and value co-creation of women entrepreneurs.

Author(s) and year	Focus/context	Key findings	Relationship to value co-creation of women entrepreneurs
Jack et al. (2010)	Entrepreneurial networking processes	Networks act as a foundation for information flow, trust, and collaboration.	Women entrepreneurs leverage social and business ties to co-create value with customers and partners.
Aldrich and Cliff (2003)	Family embeddedness in entrepreneurship	Social networks influence access to resources and the exploitation of opportunities.	Women rely on familial and community networks to mobilize support and co-develop value propositions.
Greve and Salaff (2003)	Stages of network development	Entrepreneurs activate different networks during venture creation.	Women’s relational approach fosters co-creation through long-term trust-based exchanges.
Ozgen and Baron (2007)	Social networks and opportunity recognition	Networks enhance exposure to novel ideas and resources.	Women’s networks stimulate innovative value co-creation through shared insights and mentoring.
Hoang and Antoncic (2003)	Conceptualizing social capital in entrepreneurship	Networks provide structural, relational, and cognitive capital.	Women entrepreneurs co-create value through trust, reciprocity, and shared learning in networks.
Prasad and Nair (2024)	Conceptualizing social capital in entrepreneurship	Networks provide structural, relational, and cognitive capital.	Women entrepreneurs co-create value through trust, reciprocity, and shared learning in networks.
Al-Dajani and Marlow (2013)	Women’s social enterprises in developing contexts	Networks serve as social support and resource-sharing mechanisms.	Through social networks, women co-create social and economic value in community-oriented ventures.

**Figure 1:**
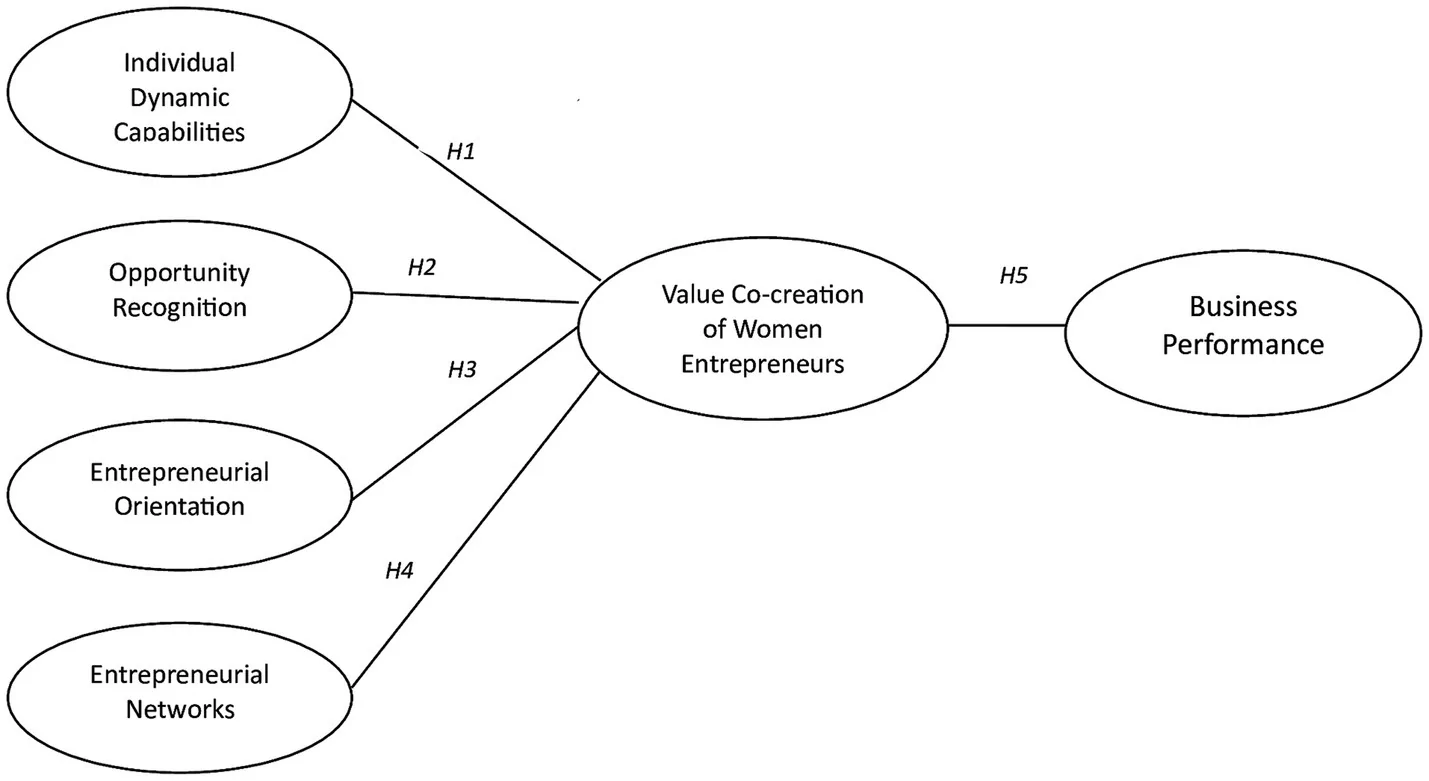
The proposed theoretical framework.

## 3 Literature review

### 3.1 Value co-creation

Historically, value co-creation has been essential for business survival, innovation, and growth (Ronchi et al., 2021). Businesses, particularly SMEs, are increasingly collaborating with stakeholders, including customers, suppliers, and employees, to co-create value (Sarker et al., 2012; Karagouni and Protogerou, 2015). They collaboratively create unique experiences through interactions and feedback, especially when developing new products, services, or launching new businesses (Prahalad and Ramaswamy, 2004; Alves et al., 2016; Fan and Luo, 2020). Value co-creation involves firms and customers collaborating to generate value, enabling customers to influence the product or service experience to meet their specific needs (Abu Bakar et al., 2007; Prahalad & Ramaswamy, 2004). The primary objective is to foster collaboration between entrepreneurs and customers in the development of products and services by gathering valuable input to enhance growth and competitiveness. Although the role of customers in value co-creation is sometimes misunderstood (Chatterjee et al., 2022), Prahalad and Ramaswamy (2004) clarified this misconception by explaining value co-creation through four key pillars: dialogue, access, risk, and transparency. These pillars emphasize the interaction between firms and customers and are essential for determining what should be co-created. They also provide a practical framework to accommodate diverse individuals with different interests, given their varied needs. Additionally, businesses involved in value co-creation can gain advantages over their competitors in various ways.

To further strengthen the theoretical grounding of value co-creation, it is essential to acknowledge seminal contributions beyond Prahalad and Ramaswamy (2004). For instance, Grönroos (2011) offers a critical analysis of value co-creation within the service logic perspective, emphasizing that value is not created by firms alone but co-created through the customer’s value creation processes. This expanded conceptualization broadens the understanding of how entrepreneurs, including women, engage customers as active partners in shaping value outcomes.

At this juncture, it becomes crucial to ask: Why is a gender-specific lens on value co-creation necessary? While traditional literature has largely focused on firm-centric or market-driven views, emerging studies highlight that women entrepreneurs approach co-creation differently—drawing on relational, empathetic, and community-oriented values (Ahl, 2006; Calás et al., 2009; Eddleston and Powell, 2008). From a feminist entrepreneurship perspective, these relational orientations are not merely social preferences but strategic practices that shape how women build trust, share resources, and mobilize collective knowledge within entrepreneurial ecosystems (Marlow and McAdam, 2013; Jennings and Brush, 2013). Integrating Grönroos’s (2011) service logic insights with feminist entrepreneurship perspectives provides a multidimensional view of value co-creation—where the co-production of value is both economic and relational. This perspective reframes value co-creation as both an economic and a social process—where collaboration and inclusivity function as sources of innovation and resilience, particularly for women-led enterprises operating in constrained environments (Brush et al., 2009; Henry et al., 2016).

First, value co-creation enhances performance by mitigating risks, reducing production costs, fostering innovation, and increasing employee turnover (Zhang and Chen, 2008). Second, value co-creation can lead to the generation of new ideas that provide entrepreneurs a competitive advantage by leveraging customers’ innovativeness, creativity, and capabilities (Prahalad & Ramaswamy, 2004a; Lorenzo-Romero et al., 2014; Giner and Peralta-Rillo, 2016).

### 3.2 Women entrepreneurship: an overview

Globally, women account for one-third of high-growth entrepreneurs and one in three innovative entrepreneurs targeting regional and international markets (GEM, 2021). They are an increasing economic force that fosters gender inclusiveness, especially in emerging economies (OECD, 2004; Okafor and Mardi, 2010). Although progress has been made in developed economies, women’s participation in entrepreneurship in the United States has increased by 1.5 percent over the past 15 years, making up 30 percent of all entrepreneurial activities (American Express, 2019). In the UK, for example, women entrepreneurs have experienced significant growth, with a start-up increase of 5.1 percent, surpassing Germany’s 4.8 percent. This marks a notable milestone in the strides women entrepreneurs have taken towards achieving gender inclusivity in business and other economic sectors.

In developing economies, the MIWE (2020) highlights that growth-oriented women entrepreneurs are more prevalent in countries such as Nigeria, Malawi, Botswana, Uganda, and Ghana. In these countries, women account for 30 to 40 percent of all businesses, despite facing structural and socio-cultural barriers that limit their entrepreneurial opportunities. The report further notes that between 30 and 40 percent of women in upper-middle, lower-middle, and low-income countries, such as Ecuador, Chile, Nigeria, India, Botswana, Egypt, Angola, and Uganda, engage in entrepreneurial activities (MIWE, 2020). In developing economies, the MIWE (2020) reveals that growth-focused women entrepreneurs are more prevalent in countries such as Nigeria, Malawi, Botswana, Uganda, and Ghana. In these nations, women entrepreneurs make up 30 to 40 percent of all businesses, despite structural and socio-cultural barriers that limit their access to entrepreneurial opportunities. The report noted that 30 to 40 percent of women in upper-middle, lower-middle, and low-income countries, such as Ecuador, Chile, Nigeria, India, Botswana, Egypt, Angola, and Uganda, are involved in entrepreneurial activities (MIWE, 2020).

Previous studies have shown extensive research on women’s involvement in entrepreneurship and economic development (Okafor and Mardi, 2010; Ali et al., 2021; Banu et al., 2021; Anderson and Ojediran, 2022). However, a gender gap in women entrepreneurs persists across all continents (Hailemariam et al., 2022). Consequently, Adachi and Hisada (2017) suggested that if these widening gender gaps in entrepreneurship are not properly addressed, efforts to support women entrepreneurs will result in minimal social change and could negatively impact their overall performance.

To further strengthen the theoretical grounding of women’s entrepreneurship research, it is essential to integrate seminal gender-aware frameworks. For instance, Brush et al. (2009) developed a gender-aware framework for women’s entrepreneurship that emphasizes the importance of context, identity, and social structures in shaping entrepreneurial behavior and opportunity recognition. This framework broadens the understanding of how gender influences entrepreneurial processes, encouraging more inclusive policies and research agendas that recognize women’s unique contributions to innovation and economic development.

Therefore, supporting women entrepreneurs could help close the gender gap in entrepreneurship and promote collaboration among stakeholders to support women’s innovative entrepreneurial initiatives.

### 3.3 Value co-creation of women entrepreneurs

Value co-creation represents a paradigm shift in entrepreneurship, where value is built collaboratively rather than by a single actor. It highlights the participatory role of customers, employees, suppliers, and communities in shaping entrepreneurial results (Vargo and Lusch, 2004; Prahalad and Ramaswamy, 2004). Within the context of women entrepreneurs, value co-creation involves actively engaging with networks and relationships to develop solutions, share resources, and boost their innovation capacity. Previous research has increasingly recognized the value of co-creation as a key approach for women entrepreneurs, especially those managing small and medium-sized enterprises (SMEs), to work with stakeholders and generate and capture value. Instead of a one-time event, co-creation is an ongoing, relationship-focused process that develops throughout the entrepreneurial journey, from identifying opportunities to expanding the business (Prahalad and Ramaswamy, 2004; Vargo and Lusch, 2016). During the opportunity recognition phase, women entrepreneurs often collaborate with customers to identify unmet needs and develop market-ready solutions. Through participatory design and continuous feedback, they reduce uncertainty and improve product-market fit.

More importantly, value co-creation functions as a growth mechanism that enables women entrepreneurs—particularly those in early-stage and resource-constrained SMEs—to overcome financial, informational, and institutional limitations. By engaging customers, partners, and networks in shared problem-solving, women entrepreneurs mobilize external expertise, reduce innovation costs, and access new markets, thereby enabling business growth by improving product-market alignment, enhancing customer loyalty, and acquiring new knowledge and technologies (Payne et al., 2008). This strategic collaboration transforms value co-creation from a relational activity into a deliberate capability-building process that enhances long-term competitiveness and resilience.

Co-creation also appears in how women entrepreneurs mobilize resources through networks, using social ties, cooperatives, and women’s groups to access knowledge, funding, and markets, strengthening collective empowerment and trust (Aldrich and Cliff, 2003). By functioning as a strategic mechanism for overcoming scarcity, co-creation allows entrepreneurs to turn relational assets into tangible growth outcomes, bridging the gap between limited resources and ambitious market goals (Chatterjee et al., 2022). This development enables women entrepreneurs to be viewed not as isolated individuals, but as network orchestrators who blend diverse knowledge, experience, and resources to create shared value. Through trust-based collaboration and community engagement, they overcome barriers such as limited financial access, gender barriers, and market exclusion (Al-Dajani and Marlow, 2013). Co-creation thus becomes both a strategic mechanism for competitiveness and a social process of empowerment, with multiple stakeholders working together to achieve value creation and a competitive advantage.

Similarly, women entrepreneurs benefit from value co-creation through various interconnected aspects that boost their success and growth. Central to this is leveraging collaborative networks and social capital, where women utilize both formal and informal ties—such as family, peers, and professional groups—to access key resources, market insights, and support that sustain their businesses (Shaw, 2006). Involving customers and gathering their feedback helps women design products and services that are aligned with market needs, fostering loyalty and innovation (Prahalad and Ramaswamy, 2004). Additionally, exchanging knowledge and resources helps overcome financial and institutional obstacles, as women engage in mentorship, collaboration, and peer learning to enhance skills and competitiveness (Brush et al., 2019).

Co-creation also empowers women and creates mutual benefits by positioning them as equal participants in economic and social processes, strengthening confidence, legitimacy, and agency in male-dominated markets (Al-Dajani and Marlow, 2013). Many women entrepreneurs also focus on community and social impact, embedding social goals such as job creation, inclusion, and local development into their businesses, thereby linking entrepreneurship to broader societal change and sustainable development. Together, these elements underscore the importance of collaboration, inclusivity, and shared value in supporting women’s entrepreneurial success across different contexts.

Prahalad and Ramaswamy (2004) introduced the DART model, which uses four key dimensions—Dialogue, Access, Risk Assessment, and Transparency—to provide a core framework for understanding value co-creation. This concept emphasizes the inclusive and relational aspects of women entrepreneurs’ business efforts. Women entrepreneurs often use dialogue, defined as two-way communication and active involvement, to build connections with stakeholders and customers, co-design products, improve services, and gather honest feedback. In addition to ensuring that offerings match customer values, this conversation also fosters trust. Access shows how female entrepreneurs often democratize opportunities, resources, and information mainly through digital tools, social media, and community networks by allowing clients to engage actively with the company. As such, women entrepreneurs frequently face societal and market uncertainties, risk assessment is essential for their success and growth (Prahalad and Ramaswamy, 2004; Vargo and Lusch, 2016; Ali et al., 2021). Therefore, women entrepreneurs overcome structural barriers and advance social and economic development through collaboration, mutual benefit, and shared learning. The framework highlights the importance of co-creation in understanding women’s entrepreneurship in today’s world.

The table summarizes prominent definitions of value co-creation from the literature. It contextualizes their relevance to women entrepreneurs, particularly in small and medium-sized enterprises where collaboration, empathy, and customer interaction are critical for innovation and growth. In this research, the concept of value co-creation of women entrepreneurs is operationalized as a collaborative process where women work with customers and other stakeholders to jointly develop products, services, and experiences that create mutual benefits. This process includes sharing resources, knowledge, and feedback through ongoing interaction, dialogue, and relationship-building. Such engagement helps women entrepreneurs improve innovation, market relevance, and business sustainability, while also empowering stakeholders by fostering participation and inclusion.

### 3.4 Antecedents of value co-creation of women entrepreneurial

This section summarizes the antecedents of value co-creation by women entrepreneurs (VCW), which have been empirically tested in previous research. There are four categories of antecedents: individual dynamic capabilities, opportunity recognition, entrepreneurial orientation, and entrepreneurial networks.

#### 3.4.1 Individual dynamic capabilities

The concept of individual dynamic capabilities (IDC) expands the firm-level framework of dynamic capabilities developed by Teece (2007) to integrate the microfoundations of entrepreneurial action. It emphasizes how individuals’ ability to sense, seize, and reconfigure resources helps them adapt and generate value in changing environments. IDC includes the cognitive, behavioral, and relational capacities that enable entrepreneurs to identify opportunities, mobilize resources, and transform organizational routines (Mudalige et al., 2019; Hernández-Linares et al., 2021, 2024). In entrepreneurship research, IDC is increasingly viewed as essential for explaining how founders, especially women entrepreneurs, navigate uncertainty and foster innovation despite resource limitations.

Previous research has highlighted the importance of Individual Dynamic Capabilities (IDC) in creating value for both businesses and customers. IDC can be understood as the entrepreneurial ability to integrate, develop, and reorganize internal and external resources and competencies to respond to and influence a rapidly changing business environment (Zahra et al., 2006; Katkalo et al., 2010; Teece, 2010, p. 692). At the individual level, IDC can help entrepreneurs adapt and develop new internal processes, as well as engage new stakeholders to support the creation, retention, and sustainability of the new values required in times of change. This, in turn, triggers market disruptions that directly influence business outcomes (Teece, 2012).

Women entrepreneurs and stakeholders can leverage dynamic capabilities (DC) to build a shared understanding of customer preferences, business challenges, and technological advancements. They can further validate and improve these insights, then take action by reallocating resources and adjusting activities to promote ongoing innovation and adaptation (Teece, 2014). Women entrepreneurs, especially those running small and medium-sized enterprises (SMEs), often face environments with institutional and social obstacles, such as limited access to finance, smaller networks, and role-based expectations (Costin et al., 2021). Despite these hurdles, studies indicate that women utilize their sensing abilities through market scanning, customer feedback, and community involvement to identify niche opportunities (Feranita et al., 2025). Their ability to seize opportunities is reflected in strategic networking, creative problem-solving, and flexible resource mobilization (Putritamara et al., 2023), while transformation skills support ongoing learning and business adaptation during technological shifts and crises like COVID-19 (Hernández-Linares et al., 2021). This flexibility helps build resilience, foster innovation, and maintain competitiveness (Teece, 2018).

Eisenhardt and Martin (2000) argue that consistent routines using dynamic capabilities can create opportunities for new engagement within the entrepreneurial cycle by modifying established capabilities. This approach fosters open discussions about ideas that lead to value co-creation and long-term profitability. Teece (2014, p. 332) classified dynamic capabilities into three main clusters, which are significant for contextualizing the value co-creation of women entrepreneurs. They involve identifying, developing, co-developing, and assessing technological opportunities in relation to customer needs (sensing), mobilizing resources to address those needs and opportunities, capturing value from them (seizing), and ensuring continued renewal (transforming).

Recent research on gender-focused extensions of the theory of dynamic capabilities suggests that women entrepreneurs show distinct patterns in developing IDC because of their embeddedness in relational and community networks, which influence their opportunity recognition and decision-making processes. Scholars argue that these gendered dynamics do not necessarily weaken IDC but instead diversify how they are expressed, often highlighting collaboration, empathy, and social value co-creation (Costin et al., 2021). However, the literature also identifies a capability gap where structural constraints limit the full translation of IDC into firm-level outcomes, especially in developing economies (Feranita et al., 2025).

Therefore, women entrepreneurs should seek both internal and external feedback to maximize their return on investment and enhance their reputation in the market, particularly as competition in the global marketplace intensifies. By positioning themselves effectively, they can develop the right products and target specific market segments, utilizing DC integration to focus on their offerings. Therefore, this approach allows them to identify and seize opportunities rather than cling to outdated practices, ultimately fostering growth and competitiveness (Teece, 2012). Hence, IDC relies heavily on individual entrepreneurs to identify and explore market opportunities, enabling the development of effective response strategies (Teece et al., 2016). Based on the above arguments, this study aims to propose the following hypothesis:

*H1*. Individual dynamic capability is positively associated with value co-creation of women entrepreneurs.

#### 3.4.2 Opportunity recognition

Recognizing and selecting the right opportunities for new businesses is crucial for successful entrepreneurs. Over the years, these ideas have sparked intense debate among researchers studying Entrepreneurship (Shepherd and Patzelt, 2018). It is widely believed that identifying high-potential opportunities can significantly enhance a firm’s profitability, growth, and competitive edge (Stevenson, 1983; Venkataraman, 2019). This has led scholars to propose models that serve as the basis for opportunity recognition, which include prior knowledge, social networks, entrepreneurial alertness, and traits (Hills et al., 1997; Shane and Venkataraman, 2000). Since then, researchers have continued to develop models for identifying and creating opportunities, using hypotheses from various fields like cognitive science, psychology, and economics, which sometimes complement each other (Schwartz, 2000; Ardichvili et al., 2003).

Further research has continued to provide insights into how entrepreneurs recognize and frame new ways to create mutual value through interactions with stakeholders, customers, and communities (Dimov, 2011). From a value co-creation perspective, opportunities are identified not only for their potential profitability but also for their capacity to generate shared benefits through collaboration and resource sharing (Prahalad and Ramaswamy, 2004). This relational approach aligns with the entrepreneurial behaviors of women, who often focus on social value, relationship-building, and customer engagement when identifying opportunities (Brush et al., 2019).

While these efforts have significantly improved the understanding of opportunity recognition, they do not provide a comprehensive explanation of the process because each perspective primarily focuses on a single area. For example, previous research has examined self-perceived alertness, social networks, prior knowledge, and experience as key factors in successful opportunity recognition (Shane and Venkataraman, 2000). However, focusing on one specific aspect can lead to less attention being given to other important fields that are also relevant. This is because recognizing an opportunity is often viewed as a situation that can be exploited, but it also involves risks and uncertain outcomes (Bakar et al., 2016; Shane and Venkataraman, 2000).

As a result, opportunity recognition becomes a key part of entrepreneurship that needs further clarification. It refers to entrepreneurs’ ability to identify opportunities and fulfill market demands by innovatively combining resources and leveraging them to increase business profitability (Ardichvili et al., 2003). Researchers in entrepreneurship are primarily interested in understanding why and how specific individuals can identify and pursue opportunities, leading them to start new businesses. In contrast, others have yet to achieve this. Among the factors supporting opportunity recognition are prior knowledge of the market, understanding of customers’ needs, working experience, creativity, and social networking (Shane and Venkataraman, 2000; Ardichvili et al., 2003; Ozgen and Baron, 2007; Shepherd and Patzelt, 2018). Thus, women entrepreneurs often discover opportunities through feedback from networks, mentors, and customers. This helps integrate different perspectives and encourages creative solutions (Henry et al., 2016; Mitchelmore and Rowley, 2013). These cooperative relationships enhance venture legitimacy and sustainability in addition to improving opportunity recognition, particularly in settings with limited resources (Davidsson, 2015; Zahra et al., 2006).

Based on the foregoing, opportunity recognition forms the basis for value co-creation among women entrepreneurs. By identifying and shaping opportunities collaboratively with customers, partners, and communities, women entrepreneurs turn insights into co-created innovations and shared socio-economic value. In light of this argument, this study proposes the following hypothesis:

*H2*. Opportunity Recognition is positively associated with value co-creation of women entrepreneurs.

#### 3.4.3 Entrepreneurial orientation

One of the main areas of research in entrepreneurship literature is the concept of entrepreneurial orientation. Entrepreneurial orientation defines the firm-level strategy that firms use to enact their organizational purpose, sustain their vision, and create competitive advantage(s). The concept has become a widely studied area, especially in SMEs, innovation, and women entrepreneurship (Hashimu, 2018; Iqbal et al., 2021). Previous research has shown that EO is strongly linked to value co-creation, innovation, and firm performance (Liu and Huang, 2020).

Entrepreneurial orientation helps individuals and organizations identify and launch the joint creation of products and services. In the EO literature, the unidimensional view of EO encompasses risk-taking, innovativeness, and proactiveness, whereas the multidimensional view includes these elements, as well as competitive aggressiveness and autonomy (Miller, 2011; Lumpkin and Dess, 1996). However, most EO research has used a one-dimensional view, focusing on innovativeness, risk-taking, and proactiveness (Gupta and Gupta, 2015).

Innovativeness in EO reflects the entrepreneurial willingness to innovate within the business scope. Proactiveness relates to the entrepreneurial ability to anticipate and respond to future market needs, thereby gaining a competitive edge over rivals (Jiang et al., 2018). Risk-taking involves the ability to invest resources in actions that are likely to fail, as the outcomes of such efforts are usually unpredictable (Jiang et al., 2018). Empirical studies have shown that EO can help organizations and entrepreneurs define business objectives, sustain their vision, and develop strategies to gain a competitive advantage (Mozumdar et al., 2022). When women entrepreneurs proactively take risks to drive innovation, they increase their chances of engaging in value co-creation. This, in turn, fosters business growth and improves performance.

Research has indicated that women entrepreneurs integrate entrepreneurial orientation (EO), comprising innovativeness, proactiveness, and risk-taking, into value co-creation through collaborative, socially embedded practices that extend beyond traditional competitive approaches rather than relying on individualistic or aggressive entrepreneurial behaviors. They demonstrate collaborative innovation by involving stakeholders in participatory design and feedback-based processes. They also show socially embedded proactiveness by leveraging strong relational networks to anticipate the needs of stakeholders and the community (Brush et al., 2019).

Therefore, women entrepreneurs integrate EO into value co-creation by translating EO dimensions into relational, incremental, and network-based practices. Innovativeness and proactiveness appear as dialogic, user-involved design; risk-taking is managed through shared feedback and pilot approaches; networks and ecosystems act as key enablers; and capability gaps and institutional barriers shape the form and effectiveness of the integration. In light of this argument, this study proposes the following hypothesis:

*H3*. Entrepreneurial orientation is positively associated with value co-creation of women entrepreneurs.

#### 3.4.4 Entrepreneurial networks

Entrepreneurial networks are crucial in helping businesses succeed in a fast-changing and competitive environment. These networks allow entrepreneurs to connect with other business experts, sharing knowledge, resources, and information (Abbas et al., 2019). It comprises groups of entrepreneurs dedicated to engaging in business activities, fostering social relationships, and connecting various actors, activities, and resources (Zardini et al., 2023). Prior research has viewed entrepreneurship as a socially embedded activity rather than an individual endeavor, emphasizing the ways in which relationships with family, peers, suppliers, and institutions impact value co-creation and innovation through social networks (Vrontis et al., 2020; Zardini et al., 2023).

Research on women entrepreneurs shows gender-specific patterns in how they access and use networks. Women often encounter obstacles to forming broad financial and institutional connections and tend to depend on close, trust-based relationships (Jennings and Brush, 2013). In various entrepreneurial settings, networks act both as active tools and supportive structures that promote opportunity recognition, information sharing, and value creation. Women entrepreneurs who actively participate in these networks overcome business challenges, generate shared innovative value, and guide businesses toward sustainable collaboration, growth, and performance (Welsh et al., 2018). It also promotes the development of business networking as a key to resource acquisition and integration, enabling stakeholders to collaborate in the co-creation of products and services. Previous research has highlighted the vital role that entrepreneurial networks play in fostering shared value, particularly in the context of women’s innovation. These networks support collaboration, which in turn enhances customer responsiveness to the co-creation of value (Welsh et al., 2018). In essence, the networks of women entrepreneurs function not merely as supportive backdrops but as dynamic mechanisms that enable opportunity discovery, knowledge sharing, and value co-creation across diverse entrepreneurial contexts.

In their research, Nusantara (2024) proposed that businesses leveraging entrepreneurial networks have access to a wide range of resources, skills, knowledge, and capabilities. It further helps women entrepreneurs connect with various stakeholders within a collaborative network space. This network sphere includes direct relationships between stakeholders and platform networks where value is co-created (Rasmussen, 2021). Furthermore, examined the mechanisms by which SMEs can leverage their networks to become network orchestrators, thereby creating local value and building strong business relationships. Their findings suggest that value can be created through stakeholder collaboration, highlighting the importance of trust-based relationships in enhancing value among local SMEs.

While our framework posits entrepreneurial networks as a direct antecedent (H4), their role may be more complex. Future research should explore whether networks also act as a moderating variable, strengthening the relationship between other antecedents (e.g., individual dynamic capabilities) and value co-creation, or even as a mediating variable that channels the effect of other resources. This expanded perspective would allow for a more nuanced understanding of how network structures and dynamics influence the value co-creation process among women entrepreneurs.

Therefore, entrepreneurial networks are key social capital systems that give women access to knowledge, legitimacy, and resources, which are often limited by gender-based barriers in entrepreneurship. Through these networks, women entrepreneurs build collaborative relationships with stakeholders, fostering mutual learning and innovation. These relationships foster mutual trust, facilitate the sharing of information, and support the co-creation of products and services that meet customer needs and align with social impact goals. Furthermore, women’s networking behaviors, characterized by reciprocity, empathy, and long-term commitment, help create sustainable ecosystems for value co-creation. In short, entrepreneurial networks boost women’s ability to innovate and grow, while also acting as drivers for inclusive value creation, thereby strengthening the connection between social embeddedness and entrepreneurial success. In light of the aforementioned literature, this study proposes the following hypothesis:

*H4*. Entrepreneurial network is positively associated with value co-creation of women entrepreneurs.

### 3.5 The consequences of value co-creation of women entrepreneurial

This section examines the relationship between the value co-creation of women entrepreneurs and business performance from the management and marketing perspectives.

#### 3.5.1 Business performance

In the context of often resource-constrained women-led ventures, the ultimate justification for engaging in value co-creation is its expected positive impact on business performance, which is critical for survival and growth (Ratten and Jones, 2021; Welsh et al., 2018). Measuring business performance is challenging because many factors affect its size and direction (Taghian and Shaw, 2010; Zulkiffli, 2014). Although the concept is widely accepted in scholarly literature, there is a lack of consensus on how it has been operationalized and measured (Venkatraman and Ramanujam, 1985; Taouab and Issor, 2019). This creates an absence of an operational definition upon which most researchers agree. As a result, various scholars have defined the concept using either objective or subjective terms or both. Taouab and Issor (2019) defined business performance “as the overall index of the ability of the firm to satisfy its stakeholders, measured in terms of financial as well as operational indicators, using primary data to measure ‘subjective business performance,’ secondary data to measure ‘objective business performance,’ or both.”

Thus, both firm and individual entrepreneurs set financial and non-financial indicators to provide information on the level of accomplishment, using both subjective and objective results (Vij and Bedi, 2016). Basically, businesses are assessed using both short- and long-term indicators to measure failure and success and to meet their goals in terms of financial and non-financial measures. In particular, for women entrepreneurs operating within constrained ecosystems, value co-creation serves as a mechanism to enhance competitiveness and long-term viability by integrating customer feedback, fostering innovation, and leveraging networks for performance improvement (Ratten and Tajeddini, 2018; Ribeiro et al., 2021).

While there are few studies that explicitly show a positive relationship between VCC and business performance, others offer indirect evidence, such as “market share, new product introduction, product quality, marketing effectiveness, manufacturing value-added, and other technological efficiency measures within the business performance domain” (Venkatraman and Ramanujam, 1985). This research carefully examines the relationship between women entrepreneurs’ value co-creation and business performance as a construct and outcome variable. Consistent with previous studies (Venkatraman and Ramanujam, 1985; Zulkiffli, 2014), this paper categorizes business performance into two types of performance:

1. Financial/Objective variable measuring financial performance and
2. Non-financial/Subjective variable measuring non-financial performance.

Financial objectives consist of commonly used accounting measures for evaluating business performance, such as earnings per share, return on assets (ROA), return on investment (ROI), number of customers, sales value, sales volume, and turnover (Venkatraman and Ramanujam, 1985; Taghian and Shaw, 2010; Zulkiffli, 2014; Taouab and Issor, 2019; Kim et al., 2020). However, researchers have strongly criticized these accounting measures because they overlook other aspects of business performance, such as operational performance, in favor of an economic dimension (Mohamad et al., 2022; Venkatraman and Ramanujam, 1985). As a result, few studies examine the relationship between value co-creation by women entrepreneurs and business performance. Therefore, this research aims to provide evidence on how the value co-creation of women entrepreneurs impacts business performance.

Similarly, research on financial performance has highlighted the significance and validity of financial objectives within a business’s goal framework. In their study, Peters and Waterman (1982) discovered that 36 American companies achieved exceptional performance scores across six measures: average return on market-to-book value, compounded asset growth, average turnover growth, average return on total capital, average return on sales, and average return on equity. Kyere and Ausloos (2021) examined the relationship between value co-creation and financial performance, using return on assets and Tobin’s Q. Their findings, based on 252 registered firms listed on the London Stock Exchange, were mixed.

Furthermore, Pantea et al. (2014) used return on investment and return on equity as financial performance indicators to evaluate firm growth, firm size, human resources, capital intensity, and corporate social responsibility (CSR) across 55 Romanian firms listed on the Bucharest Stock Exchange. These findings align with previous studies focused on publicly registered firms (Pantea et al., 2014; Kyere and Ausloos, 2021). Accordingly, this research emphasizes three crucial financial performance metrics: return on investment, return on assets, and the current ratio.

Consequently, researchers often use non-financial measures to assess business performance in areas such as new product launches, market share, product quality, marketing effectiveness, and technological efficiency, relying on primary data (Venkatraman and Ramanujam, 1985; Vij and Bedi, 2016). In their study, Selvam et al. (2016) developed a subjective model for assessing business performance that encompasses factors such as the firm’s market value, customer satisfaction, employee satisfaction, growth, profitability, environmental audits, corporate governance, and social performance. In a microbusiness setting, using non-financial variables is the most effective way to measure business performance (Mohamad et al., 2019; Hashimu, 2018). When firms employ subjective measures, they focus on key indicators such as employee and customer satisfaction, product innovation, product quality, and process innovation (Vij and Bedi, 2016). These indicators represent the non-financial aspects of performance measurement.

Additionally, researchers have highlighted that data obtained through subjective methods are considered reliable for assessing business performance (Vij and Bedi, 2016; Hashimu, 2018). Although existing research has demonstrated relationships between value co-creation and business performance (Kim et al., 2020), further investigation is necessary to deepen the understanding of the relationships between these constructs. Specifically, research on the direct effects of value co-creation within the context of women entrepreneurship (VCCWE) is still lacking. This study aims to address these gaps by reviewing literature on the relationships between VCW and business performance. The literature revealed that prior studies have not adequately measured the relationship between VCW and subjective business performance, particularly in the context of business performance.

Therefore, women entrepreneurs who co-create through strong relationships and networks gain access to valuable resources, including knowledge, market access, and social capital, which in turn enhance innovation, market fit, and operational resilience. These resources are then utilized to enhance firm-level outcomes, such as customer retention, sales growth, and survival (Welsh, 2021; Ribeiro et al., 2021). In light of the aforementioned literature, this study proposes the following hypothesis:

*H5*. Business performance is positively associated with value co-creation of women entrepreneurs.

### 3.6 Proposed theoretical framework

The extensive review of literatures done despite the conceptual framework as shown below in Figure 1.

The proposed conceptual framework, which is derived from the extensive literature, can be empirically tested in a qualitative approach by conducting an interview or focus group among Women Entrepreneurs. This approach will help to reassess the constructs’ dimensionality and to examine the antecedents of Individual Dynamic Capabilities (H1), Opportunity Recognition (H2), Entrepreneurial Orientation (H3), and Entrepreneurial Networks (H4) on the Value Co-creation of Women Entrepreneurs. Additionally, it will examine the consequence of the Value Co-creation of Women Entrepreneurs on Business Performance (H5) from the entrepreneurs’ perspective. In addition, the validation and development of instrumentation at this stage will enhance the robustness of measurement and the model for the future agenda of quantitative research approaches.

## 4 Research agenda and methodological implications

The proposed conceptual framework provides a foundational theory; however, its contribution must be validated and extended through rigorous empirical inquiry. This section outlines a research agenda and the associated methodological implications to guide future studies in this domain. A critical first step involves the measurement of the core constructs. While established scales exist for entrepreneurial orientation (Lumpkin and Dess, 1996), opportunity recognition (Shane and Venkataraman, 2000), and dynamic capabilities (Teece, 2007), the construct of “value co-creation of women entrepreneurs” (VCW) requires careful contextualization. Future research should develop and validate a multidimensional scale for VCW, potentially adapting items from general value co-creation scales (e.g., Grönroos, 2011; Ranjan and Read, 2016) to reflect the relational, trust-based, and often community-oriented practices prevalent among women entrepreneurs (Brush et al., 2019). This ensures the construct is captured in a way that is both psychometrically sound and contextually relevant.

The research context for testing this framework offers rich avenues for exploration. Comparative studies across different geographic and sectoral contexts could yield significant insights. For instance, applying the framework to tech startups in Southeast Asia (e.g., Indonesia, Vietnam) would test its relevance in high-growth, digitally-enabled environments (Soedarsono et al., 2020). Conversely, investigating craft industries or social enterprises in Africa (e.g., Nigeria, Ghana) would explore its utility in resource-constrained, community-embedded settings where women entrepreneurs are highly active (Ribeiro et al., 2021; MIWE, 2020). Cross-country comparisons between developed and developing economies could also illuminate how institutional and cultural factors shape the co-creation process.

To achieve these objectives, a mixed-methods approach is highly recommended (Creswell and Plano Clark, 2017). An initial qualitative phase, involving in-depth interviews and focus groups with women entrepreneurs, would be invaluable for refining the VCW construct, understanding the nuances of the proposed relationships, and identifying any context-specific variables not captured in the current model. This exploratory work would provide the depth needed to finalize measurement instruments. Subsequently, a quantitative phase using large-scale surveys would allow for the statistical testing of the hypotheses (H1–H5) through structural equation modeling (SEM), assessing the overall fit of the framework and the relative strength of each path (Hair et al., 2019).

Beyond testing the direct relationships in the framework, future research should formulate and test more complex hypotheses involving mediating and moderating variables. For example, does entrepreneurial networks mediate the relationship between individual dynamic capabilities and value co-creation? Furthermore, investigating key moderators will clarify the boundary conditions of the framework. The role of culture, both national and organizational, could significantly influence how co-creation is initiated and perceived (Gbadamosi, 2019). Industry type (e.g., service vs. manufacturing) may moderate the link between co-creation and performance, while access to technology could amplify or enable co-creation activities, especially in digital platforms (Welsh et al., 2018). Testing such moderated-mediation models would substantially deepen the theoretical understanding of the value co-creation process for women entrepreneurs.

## 5 Conclusion

Value co-creation emerges as a pivotal concept in the realm of women entrepreneurship, representing a collaborative process in which entrepreneurs, customers, partners, and suppliers jointly create and realize value. Women entrepreneurs, drawing on their distinctive perspectives and experiences, play a vital role in fostering inclusive and diverse business ecosystems that promote innovation, adaptability, and sustainable growth. Through value co-creation, they strengthen their networks, enhance customer satisfaction through empathy-driven engagement, and generate long-term competitive advantages. This collaborative process is both influenced by and reinforces four critical antecedents—Individual Dynamic Capabilities, Opportunity Recognition, Entrepreneurial Orientation, and Entrepreneurial Networks—which collectively shape the mechanisms of entrepreneurial success.

The study contributes theoretically by developing a comprehensive framework that integrates these antecedents to explain how value co-creation unfolds among women entrepreneurs. This integration expands existing theories of entrepreneurship and strategic management by highlighting the interplay between internal resources and external relational factors. Moreover, it establishes the causal pathway linking co-created value to improved business performance, providing a solid foundation for future empirical investigations into co-creation dynamics across diverse entrepreneurial contexts.

From a practical perspective, the findings offer actionable insights for women entrepreneurs, support agencies, and policymakers. Women entrepreneurs are encouraged to cultivate their dynamic capabilities, refine their opportunity recognition skills, and adopt a proactive entrepreneurial orientation while actively developing strong networks that facilitate collaboration and innovation. For policymakers, these insights underscore the importance of designing targeted development programs that enhance these competencies, enabling women-led ventures to engage in more effective value co-creation and, consequently, achieve superior performance outcomes.

Overall, this study advances both theory and practice by deepening the understanding of how individual and relational factors drive value co-creation and by offering a holistic, empirically testable framework that links co-creation processes to enhanced business performance. It ultimately underscores the transformative potential of collaborative value creation as a catalyst for women’s entrepreneurial empowerment and sustainable economic growth.
